# Patterns, clusters, and transitions in U.S. state ENDS policy implementation: 2010–2020

**DOI:** 10.1371/journal.pgph.0003636

**Published:** 2024-11-13

**Authors:** Callie Zaborenko, Mike Vuolo, Jennifer L. Maggs, Jeremy Staff, Brian C. Kelly

**Affiliations:** 1 Dept of Sociology, Purdue University, West Lafayette, Indiana, United States of America; 2 Dept of Sociology, The Ohio State University, Columbus, Ohio, United States of America; 3 Dept of Human Development and Family Studies, The Pennsylvania State University, University Park, Pennsylvania, United States of America; 4 Dept of Sociology & Criminology, The Pennsylvania State University, University Park, Pennsylvania, United States of America; The University of British Columbia, CANADA

## Abstract

Electronic nicotine delivery systems (ENDS), such as e-cigarettes, have become increasingly used across the world. To respond to global public health challenges associated with vaping, governments have implemented numerous ENDS policies. This research highlights the patterns, clustering, and transitions in U.S. state ENDS policy implementation from 2010 to 2020. Policy data for tobacco and ENDS policies primarily from the Americans for Nonsmokers’ Rights Foundation (ANRF) were analyzed for the years 2010 to 2020 for all fifty states and Washington, D.C. Patterns and clusters of policies were assessed. Latent trajectories were modeled for ENDS policies across states over time. ENDS policies commonly have analogous tobacco control policies in place prior to their implementation. ENDS policies in states were commonly implemented in “bundles.” The temporal trajectories of ENDS policy implementation occurred in 3 latent forms. A majority of states were “catch-up implementers,” indicating their slow initial implementation but stronger position by the end of the period of observation in 2020. These trajectories of ENDS policies were not associated with any individual tobacco control policy in place at the start of the trajectory in 2010. The development of ENDS policies in U.S. states has been temporally and geographically uneven. Many states that had initially been slow to implement ENDS policies caught up by 2020. The implementation of policy “bundles” was common. The clustering of policies in bundles has important methodological implications for analyses, which should be considered in ENDS policy evaluations.

## Introduction

Electronic cigarettes (e-cigarettes) and other electronic nicotine delivery systems (ENDS) have increasingly been used as a mode of nicotine consumption. E-cigarettes entered markets in 2007 and rapidly became a common, albeit controversial, form of nicotine use globally. Although some health professionals have viewed e-cigarettes as a means to transition adult cigarette smokers away from tobacco [1–3], there were concerns raised regarding their use among youth [4]. Indeed, these products were quickly adopted by adolescents during the ensuing decade [5]. In 2011, only 0.6% of U.S. middle school students and 1.5% high school students had recently used e-cigarettes [6], but e-cigarettes use quickly surpassed tobacco cigarette smoking among adolescents [7]. By 2021, 2.8% of U.S. middle school students and 11.3% of high school students were currently using e-cigarettes [8]. These patterns of ENDS use among young people remain a global public health concern, particularly given that the full extent of long-term harms from e-cigarette use remains unstudied [6, 9].

Although ENDS have been advertised as containing only nicotine, flavoring, water, glycol, and glycerin, researchers have found that, in some instances, they also contained carcinogens and other toxic chemicals [10]. Although many consequences for morbidity and mortality are anticipated to arise only in the future after extended periods of use, ENDS have been associated with adverse effects on the lungs and cardiovascular system [11, 12]. Thus, even if ENDS are ultimately less harmful than smoking tobacco, early evidence indicates they still pose risks to those who use them. Individuals affected are not solely people who vape e-cigarettes; those living, working, and socializing in places without indoor vaping bans are exposed to second-hand vapors that would not be present if ENDS were included in existing clean air laws covering tobacco smoke [13]. Public policies have been essential to global reductions in tobacco use, especially among young people [14–19]. Various policies implemented to place controls on ENDS have typically been modeled on those in place for tobacco [20]. In this study, we examine the trajectory of U.S. state-level policies specific to ENDS use from 2010 to 2020. These ENDS policies include indoor bans and excise taxes, as well as restrictions on ENDS purchase ages, youth use and possession, and distribution through vending machines. Using the U.S. as a case to consider with implications for global ENDS policy shifts [16–19], we examine both temporal change and the clustering of policies and argue that understanding these patterns will be critical to research efforts to isolate the effect of any one policy, and that multiple policy studies remain a fruitful approach.

### Types of ENDS policies and their implementation in the U.S.

In response to increases in ENDS use in the U.S., states, counties, and municipalities have enacted laws to regulate these products [21]. Beyond their specific mechanisms to directly impede ENDS use, a goal of these policies is to inhibit re-normalization of nicotine use by generating obstacles to the use of vaping products. Similar laws and public health campaigns were utilized to deter the use of tobacco. Such policies have contributed to lower rates of past month use of combustible tobacco in high school students, from 39% in 1976 to 4.4% by 2021 [8]. Tobacco control policies were designed to stymie tobacco use by making it less attractive, more costly, and more difficult to access. Further, policies that restricted access to tobacco products specifically among adolescents were related to lower smoking prevalence in adulthood [22]. Individual policies deter tobacco smoking alone, but may be more effective when combined or to counterbalance the absence of another policy [22, 23].

Clean indoor air policies for combustible tobacco have a long history, with the first law being passed in Minnesota in 1975 [24]. As of January 2023, 28 states and Washington, D.C had comprehensive smoking bans for combustible tobacco (cigarettes and other forms), and others had been passed at the municipal or county level [25]. Because comprehensive indoor tobacco bans were specifically for smoking, often they did not cover more recently emerging ENDS products, which are aerosol-based and non-combustible [26]. Though the movement to include ENDS in these bans began in 2009 in one county in New York, the first state to pass clean indoor air laws that included aerosols was New Jersey, implemented in 2011 [20]. Some research has indicated that adult respondents who lived in a state with indoor vaping bans in 2016 had lower odds of using e-cigarettes [27], although others have not found such effects [28, 29]. Vaping bans may specifically disrupt adolescent ENDS initiation [30].

Excise taxes have been shown to be an effective means to reduce tobacco use [31] particularly in areas that do not have comprehensive smoking bans [23]. These findings may extend to e-cigarette use, with a 10% increase in e-cigarette prices associated with a 9.7% reduction in the number of days middle and high school students use e-cigarettes [32]. Minnesota was the first state to impose a tax on ENDS, in 2011 [33]. As of September 2022, 30 states and Washington, D.C. had enacted a tax on ENDS [34]. State taxes take three forms—tax based on specified prices, tax per milliliter of fluid, or a combination of price and fluid—and thus are often not consistent across states in a manner like per-pack tobacco taxes. Unlike the typically consistent unit of taxation on combustible cigarettes, this variation for e-cigarettes also can be a roadblock to researchers, as harmonization across jurisdictions and data sources becomes necessary [35].

Age restrictions, such as raising the minimum age of purchase to 19 or 21, are an important deterrent for youth use. Although the federal government has passed purchase of age recommendations for both tobacco (1997) and ENDS (2016, 2019), the federal government has no enforcement power since the 2000 U.S. Supreme Court case *Food and Drug Administration*, *et al*. *v*. *Brown & Williamson Tobacco Corporation*, *et al*., leaving enforcement to the states. Thus, states must pass and enforce their own age of purchase policies. In 2016, the U.S. Food and Drug Administration (FDA) recommended the minimum age of purchase for ENDS of 18 [36, 37]. However, there was a push to raise the age of purchase even further, to the age of 21 (i.e., T21 laws). At the end of 2019, the federal government passed T21 legislation which encouraged states to increase the minimum age of tobacco purchase, including both cigarettes and ENDS, to 21 nationwide. This legislation was implemented during 2020, but state implementation has not been universal and occurred in uneven fashion. Respondents who lived in a state with a minimum e-cigarette purchase age of 21 in 2016 had lower odds of using e-cigarettes [27]. Researchers found stronger T21 implementation in the Northeast and West regions of the U.S. than in the Midwest and South, and disparities were found primarily in rural areas, areas with lower college attainment, lower SES, and higher minority populations [38, 39]. Also, in areas with lower T21 implementation, there was higher adult smoking prevalence, indicating that uneven policy implementation may exacerbate existing health disparities related to tobacco use. It is estimated that these laws may reduce the lifetime use of e-cigarettes by 25% [40].

Beyond vaping bans, excise taxes, and age of purchase laws, various other laws have been implemented to constrain nicotine use. Laws that restrict purchase age often also restrict use and possession by youth; the three policies together have been called PUP (purchase, use, possession) laws. Youth who live in areas with tobacco PUP laws perceive peers to use tobacco less often and have a lower increase in smoking over time [41, 42]. However, the effectiveness of PUP policies depends on penalties when not followed [43, 44]. The most effective policies are those that disrupt sales to minors [45]. Relatedly, vending machines have historically been an easy way to obtain tobacco products without age being a barrier. Vending machines that dispense tobacco products were banned in places accessible to minors in 2010 [46]. In 2016, the FDA made it illegal nationwide to sell ENDS in vending machines except in locations where youth were not allowed such as bars [36].

Previous work has examined the uneven distribution of e-cigarette policies, showing that early implementation of e-cigarette indoor air policies started at the municipal level within a few states and then slowly became implemented at the state level [20]. Many ENDS policies were modeled on existing tobacco policies implemented in prior years. As such, multiple policies may be passed simultaneously in the form of policy bundles. Given that ENDS policies have not been implemented evenly across the United States, nor at the same time, patterns of policy implementation (such as bundling) may have implications for disparities in ENDS use across different geographic locales. As such, the aim of this paper is to characterize the implementation of e-cigarette related policies (including timing and bundling) at the state-level across the U.S during the decade from 2010 through 2020.

## Materials and methods

### Data

Policy data come primarily from the Americans for Nonsmokers’ Rights Foundation (ANRF). ANRF has collected a complete repository of tobacco- and ENDS-related ordinances and regulations across the country by date of implementation. From the restricted-access ANRF repository, we created a location-year dataset at the state level for each year. Tobacco cigarette taxes typically occur on a similar base unit (e.g., per pack), but the variety of ENDS products and approaches to state taxation prohibits this approach. We used Cotti and colleagues’ [35] harmonized approach for state-level ENDS taxes across locations using different units of taxation, which relies on retail data to identify taxes across locales. For more details on excise taxes for ENDS and their harmonization across states, please see Cotti et al. 2023 [35]. ENDS policies included comprehensive indoor vaping bans (defined as policies mandating that workplaces, bars, and restaurants are 100% vaping-free without exceptions), any ENDS purchase age restriction, youth use restrictions, youth possession restrictions, any vending machine restrictions, and any excise taxes. Tobacco policies included indoor tobacco comprehensive smoking bans, youth use restrictions, youth possession restrictions, vending machine restrictions in adult spaces, and excise taxes. We note that the data use agreement prevents the disclosure of specific policies in specific states in specific years.

### Analytic approach

State-level policy data were gathered for the years 2010–2020 for all fifty states and Washington, D.C. The e-cigarette policies were coded to represent six different types: comprehensive vaping ban (B), minimum age of sales restrictions (S), vending machine sale bans (V), youth possession ban (P), youth use ban (U), and excise taxes (T). We first assessed whether states that implemented ENDS policies had equivalent (or stronger) tobacco policies already in place at the time of ENDS policy implementation. We then examined states’ implementation of ENDS policies yearly from 2010 to 2020 by determining the number and types of policies. We visually depicted the number of policies in each state in 2011, 2015, and 2020 to highlight geographic differences in policy passage over time. Next, we calculated the percentage of each policy pattern type by year. Then, we examined which types of policies were passed simultaneously or were “bundled.” Next, we estimated latent trajectories for policies passed across states over time, utilizing the methodological standards of the approach including the identification of the most parsimonious models within the context of BIC change. Finally, we examined whether any specific tobacco control policies in 2010 were associated with ENDS policy latent trajectories.

## Results

The existing tobacco policy context established a blueprint in many states for the development of policies specific to ENDS. We examined whether states that implemented ENDS policies had a previously existing analogous policy for tobacco control. As outlined in Table 1, although there was a range of variation, states overwhelmingly previously passed analogous policies for tobacco prior to the implementation of laws specific to e-cigarettes. Comprehensive smoking bans were already well in place in all locales that implemented indoor vaping bans, and these new vaping laws were often extensions of the prior smoking bans. All but two states that implemented taxes for vaping devices and fluids had previously enhanced tobacco excise taxes above $1 per pack. Thus, the excise tax environment was already augmented beyond minimum taxes for tobacco in locales that implemented vaping related excise taxes. Of the states passing minimum age laws for e-cigarettes, all already had existing laws regarding minimum age of purchase for tobacco, and some states had already enhanced the age of purchase for tobacco above age 18 (either age 19 or 21) prior to instituting vaping age restrictions. Similarly, states modeled youth possession and youth use penalties after those in place for tobacco, with restrictions for e-cigarettes exceeding tobacco in only a few states. Lastly, with one exception, states that implemented restrictions on the sale of vaping devices from vending machines had existing laws banning sales of tobacco from vending machines even in adults only spaces, such as bars. Thus, the policy landscape for emerging e-cigarette policies is patterned after the blueprint established by prior state tobacco policies.

**Table 1:** State-level tobacco policies in place prior to e-cigarette policy implementation.

E-cigarette policy implemented between 2010–2020	# States	Number of such states with prior tobacco policy	# States
Comprehensive vaping bans (B)	16	Comprehensive smoking ban	16
Any e-cigarette excise taxes (T)	19	Tobacco state excise tax above $1/pack	17
Any e-cig age of purchase/sales laws (S)	47	Tobacco purchase/sales age laws	47
Any youth e-cig possession laws (P)	33	Youth tobacco possession laws	31
Any youth use laws (U)	18	Youth tobacco use laws	15
Any vending machine restrictions (V)	38	Tobacco vending machine bans, even in adult spaces such as bars	37

N = 50 states and Washington, D.C.

**Fig 1:**
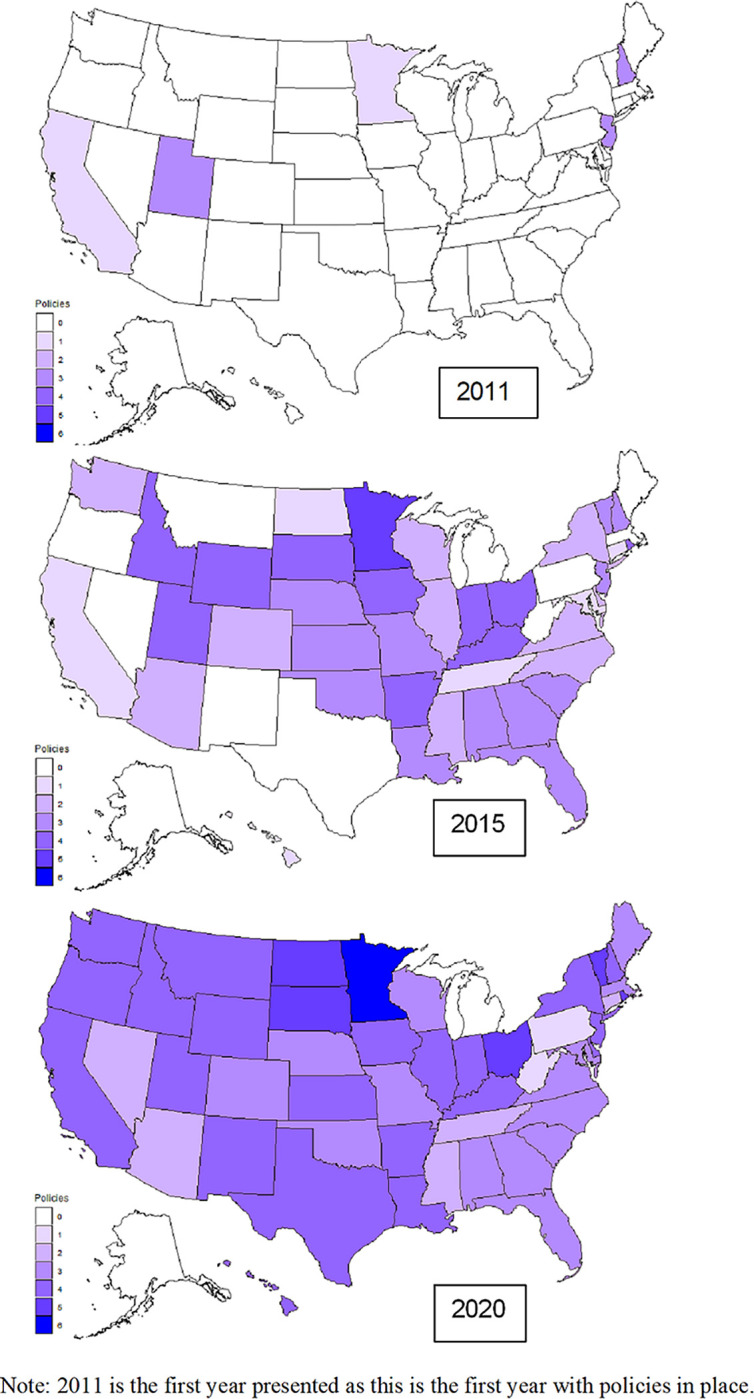
Accumulation of state e-cigarettes policies across U.S. states 2011–2020 (basemap shapefile via https://www.geonames.org/; creative commons https://creativecommons.org/licenses/by/4.0/).

As depicted in Fig 1, ENDS policies were sparse across the U.S. during 2011. In 2011, California and Minnesota each enacted a single policy, while New Hampshire, New Jersey, and Utah each simultaneously enacted three policies, albeit not the same bundles of policies. These initial states implementing early ENDS policies also tended to have strong existing tobacco control policies in place, rendering these new laws aimed at vaping an expansion of existing efforts at tobacco control. Alongside this initial implementation of policies, Fig 1 depicts the policy change over time with policy coverage by number of ENDS policies across states in 2011, 2015 and 2020. As shown, state-level ENDS policy implementation accelerated during the ensuing years. By 2015, twenty-three states had passed at least three policies focused on ENDS. At that time, the lone state with 5 policies was Minnesota, but there were 10 states that had implemented 4 policies. Only twelve states remained without any ENDS policies by that point. Two states passed a single policy during this period, West Virginia and Pennsylvania, with both passing ENDS excise taxes. By 2020, all but two states—Alaska and Michigan—had passed at least one of the ENDS policies under study. Michigan was an outlier within the Midwest region, as many of the surrounding states had more consistently implemented ENDS policies over this period.

Table 2 displays the patterns of policies implemented from 2010–2020 by patterns over time. These patterns not only show the increasing implementation of ENDS policies, but also how they accumulated across the decade to create specific policy contexts. Overall, implementation of state policies specific to ENDS increased considerably over the period of observation, signified on the top line by the transition from 100% of states with no policies in 2010 to only 4% of states (i.e., 2 states) with no ENDS policies by 2020. As noted above, in 2011, two states passed single policies, one minimum age of sales restrictions (S) and one excise taxes (T) and three other states passed policy bundles in that year—SPU (Sales; Possession; Use), SVP (Sales; Vending; Possession), and BSV (Ban; Sales; Vending). As highlighted within the table, the period from 2013 to 2016 was one of rapid shifts in such state-level policies, with 73% of states having no policies specific to ENDS in 2013. By 2016, only 12% of states had no policies. By 2020, the three most common patterns of ENDS policy implementation were 16% SVPU (Sales; Vending, Possession; Use), 12% SVP (Sales; Vending; Possession), and 10% BSVT (Ban; Sales; Vending; Taxes). States were increasingly likely to have multiple policies implemented as time passed. Yet, only one state had implemented all six policies by the end of the period. No states that passed ENDS policies during this period subsequently eliminated them, indicating a strong period of accumulation.

**Table 2:** Patterns of e-cigarette policy implementation across states (and Washington, D.C.), 2010–2020.

Pattern	2010	2011	2012	2013	2014	2015	2016	2017	2018	2019	2020
*No policies*	100%	90%	86%	73%	51%	24%	12%	6%	4%	4%	4%
*One policy*											**4%**
T: Taxes		2%	2%	2%	2%	0%	0%	4%	4%	4%	4%
S: Sales		2%	6%	10%	10%	10%	8%	6%	6%	6%	
B: Vaping ban				2%	2%	2%	0%	0%	0%	0%	
*Two policies*											**10%**
ST: Sales; Taxes											4%
SP: Sales; Possession				2%	4%	6%	8%	8%	8%	8%	4%
SV: Sales; Vending				2%	10%	14%	14%	10%	10%	8%	2%
*Three policies*											**28%**
SPT: Sales; Possession; Taxes											2%
SPU: Sales; Possession; Use		2%	2%	2%	4%	4%	4%	4%	4%	4%	4%
SVT: Sales; Vending; Taxes							2%	2%	2%	2%	4%
SVU: Sales; Vending; Use						2%	2%	2%	2%	2%	2%
SVP: Sales; Vending; Possession		2%	2%	2%	6%	16%	14%	14%	14%	14%	12%
BSV: Vaping ban; Sales; Vending		2%	2%	2%	2%	2%	2%	4%	4%	4%	4%
*Four policies*											**42%**
SPUT: Sales; Possession; Use; Taxes											2%
SVPT: Sales; Vending, Possession; Taxes							2%	4%	4%	4%	8%
SVPU: Sales; Vending, Possession; Use				2%	8%	18%	22%	22%	22%	20%	16%
BSPU: Vaping Ban; Sales; Possession; Use							2%	2%	2%	2%	2%
BSVT: Vaping Ban; Sales; Vending; Taxes							2%	2%	4%	6%	10%
BSVP: Vaping Ban; Sales; Vending; Possession				2%	2%	2%	4%	8%	6%	6%	4%
*Five policies*											**12%**
SVPUT: Sales; Vending; Possession; Use; Taxes						2%	2%	2%	2%	2%	2%
BSVPT: Vaping Ban; Sales; Vending; Possession; Taxes									2%	2%	4%
BSVPU: Vaping Ban; Sales; Vending; Possession; Use							2%	2%	2%	4%	6%
*All six policies*											**2%**

Note: Inclusive of all 50 states plus Washington, D.C., total percentage may exceed 100% due to rounding

As shown in Table 3, ENDS policies were often bundled together at the time of implementation rather than being passed one at a time. Of the 48 states that implemented ENDS policies, all but six bundled policies at least once. Two states passed two sets of policy bundles at different times. The most common policy bundle, passed thirteen times, included age of sales restrictions, vending machine sale bans, youth possession ban, and youth use ban (SVPU). Two other policy bundles were common: Age of sales restrictions and vending machine sale bans (SV) were passed together in 10 states, and age of sales restrictions, vending machine sale bans, and youth possession ban (SVP) were passed together in 9 states. Other combinations were only passed one or two times. Of note, each of the most common types of policy bundles combined barriers to youth purchasing ENDS—age of sales restrictions (S) and vending machine sale bans (V)—consistent with these being the most widely implemented strategies.

**Table 3:** Policy bundles.

Policy bundle types	Number of times implemented
SVPU	13
SV	10
SVP	9
SP	2
BSVP	2
SPU	2
BT	2
BSV	1
SVU	1
PU	1
BV	1
BPU	1

**Note:** Some states passed multiple bundles over time. Comprehensive vaping ban (B), age of sales restrictions (S), vending machine sale bans (V), youth possession ban (P), youth use ban (U), and excise taxes (T).

**Fig 2:**
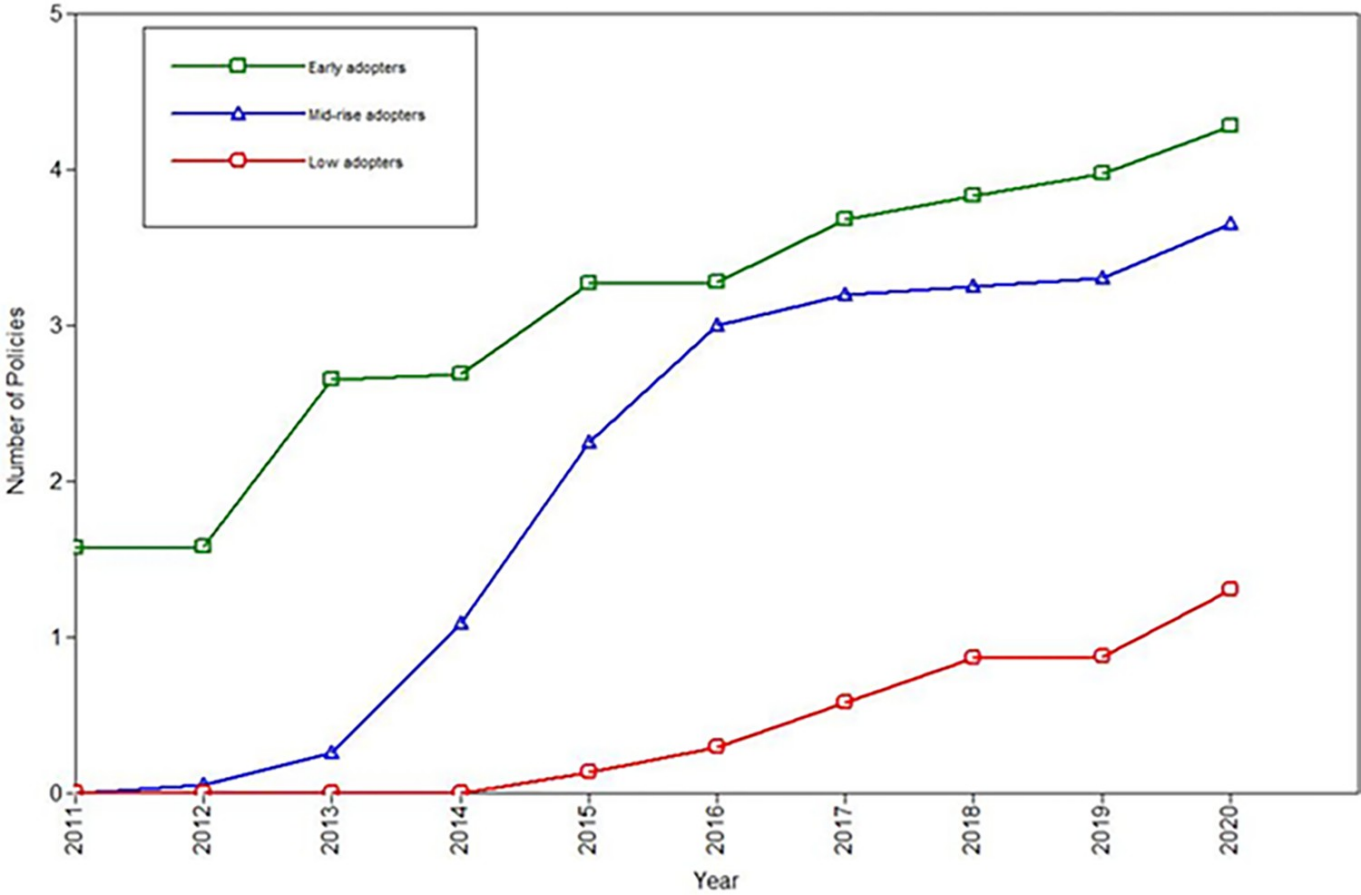
Latent classes of policy implementation.

Latent class analyses were fit to identify general trajectories of the passage of ENDS policies over time. The fit indices for the latent class analysis are depicted in Table 4. They support the 3-class solution, which is also interpretable in a straightforward manner. The latent classes themselves are graphically depicted in Fig 2. From the latent class analysis, there appear to be three primary patterns of policy implementation over time for U.S. states and Washington, D.C.: early strong implementers, catch-up implementers, and weak implementers. The early strong implementers are the seven states which passed at least one policy by 2013, at least 3 by 2017, and between 4 and 6 in 2020 (California, Idaho, Kansas, Minnesota, New Hampshire, New Jersey, and Utah). The weak implementers were the seven states which had zero policies by 2014, no more than one policy in 2017, and a maximum of 3 policies by 2020 (Alabama, Connecticut, Maine, Michigan, Nevada, Pennsylvania, West Virginia). The catch-up implementers were the remaining 36 states and Washington, D.C., which had 0 policies in 2011, and no more than 1 in 2012, but then increased the number of policies rapidly from 2013 to 2015, but they were still slightly lower than the early strong implementers by the end of the period of observation in 2020. Thus, a notable majority of states were in the “catch up” group of states that eventually had robust ENDS policies in place by 2020 but took some time to get there. Although states had overwhelmingly passed analogous tobacco policies prior to the implementation of e-cigarette laws, the trajectories were not significantly associated with any single tobacco control policy in place at the start of the time series in 2010. Thus, while tobacco policies provided a blueprint, individual tobacco laws in 2010 did not predict the subsequent temporal patterning of e-cigarette policies.

**Table 4:** Latent class analysis fit statistics.

Classes	LL	Parameters	BIC	BIC change	Entropy
1	-754.081	10	1547.481		
2	-700.875	21	1484.318	-63.163	0.937
3	-655.464	32	1436.747	-47.571	0.961
4	-655.464	43	1479.997	43.250	0.969

## Discussion

Over the course of a decade leading up to 2020, U.S. states increasingly passed policies aimed at regulating ENDS products; these were typically modeled on and implemented after existing state tobacco policies. From zero states having ENDS policies in 2010, over four-fifths of states had at least three policies by 2020. The earliest policy implementation occurred in 2011, with some states initially implementing between one and three policies. A proliferation in ENDS policy implementation ensued from 2013 to 2016. By 2020, only 2 states had no ENDS policies. The southern region of the U.S.–historically a tobacco growing region–stands out as having no laws related to ENDS during the early years of observation, and states in this region continue to have fewer policies on average than the Northeast, Midwest, and Western regions.

Often ENDS policies were bundled together during implementation, with two to four policies implemented during the same year. Only 8 U.S. states never passed a policy bundle at any point across the period of observation, highlighting how normative policy bundles were. The modeling of ENDS policies on preexisting tobacco control policies may have allowed for the efficient implementation of new regulations as bundles. Most policy bundles implemented during this period of time contained laws that would restrict age of sales and vending machine bans, although some instances included other policies as well. Thus, when policy bundles were passed, they were clearly targeting access to ENDS, and in particular restricting means of access for young people. The clustering of policy passage may not only be an efficient form of governance but may also create synergy by which multiple forms of policy strengthen each other. Bundled policy changes may also lead to greater public awareness of these legal changes. These potential synergistic effects are in need of further study.

There are some methodological considerations that need to be taken into account when examining the effects of policies due to the bundling of specific polices simultaneously. With only 8 states never passing policies in a bundle, the implications are a non-trivial matter for the future of ENDS policy evaluation. When assessing the effect of ENDS control policies, it is important to account for the broad range of e-cigarette and tobacco policies. Consider the example of age of sales restrictions: such policies were enacted a total of 40 times simultaneously in a bundle with at least one other policy, including 28 times with more than one other policy. Thus, analytically, when isolating the effect of the age of sales restrictions, it is especially important to control for multiple ENDS policies. Even for tobacco control, where the passage of policies was much more temporally and spatially variable, the effectiveness of a policy depends on what other policies are also in effect [23]. Thus, our study is an important caution for the future of ENDS policy evaluation, adding to an emerging methods-based literature on excise taxes [35]. In addition to the clustering of ENDS policy implementation, it is also notable that ENDS policies were overwhelmingly implemented with analogous existing tobacco policies, and thus the effects of ENDS policies are typically net of comparable policies for tobacco. Given that many of these same ENDS policies have been passed around the globe, such cautions regarding bundling may potentially arise elsewhere [16–19]. We encourage research in other countries to determine the extent that policies are bundled.

Within the context of the latent trajectories of policy implementation, we found that policies were implemented in a fragmentary manner across regions of the United States. Early implementers quickly passed ENDS related policies early in the period and continued to have the strongest policy contexts by the end of the period of observation. The early implementers were located in the Northeast, Midwest, and West regions. In contrast, the weak implementers were geographically diffuse states. The weak implementers were slow to begin policy implementation and continued to have weak controls on ENDS by the end of the period of observation. In contrast, although the “catch up” group (the most common latent trajectory) was slow to implement policies initially, they quickly implemented policies during the intervening years–with rapid implementation between 2013 and 2016 –and had policy contexts that were almost as strong as the early implementers by the end of the period of observation. The catch-up implementers were found in all U.S. census regions, indicating that this was a widespread and dominant trajectory of implementation. Stepping back to the international landscape, we encourage examinations of the temporal patterning of ENDS policy control across countries as well.

### Limitations

The policies under study focus on those implemented at the state-level within the U.S. We must note that policies may be implemented at the municipal or county level, and such levels of government may also engage in bundling. The purpose of our research is not to bypass these important levels of policymaking but to highlight the role of bundling at a common level of health policy passage. Furthermore, we note that enforcement also plays a key role in the effectiveness of policies. Enforcement can vary not simply between states, but within them as well. At this time, the data do not allow us to examine these within- and between-state variations in how effectively policies are enforced. It is also important to note that we do not empirically evaluate whether some combinations (i.e., bundles) are more effective than others. This is an important next step in research. For example, having a vaping ban and excise taxes (2 policies) may be better than having all three PUP laws. In this example, denormalization and economic/rational choice might be “higher quality” mechanisms of behavioral change than simply three ways that limit access. Such an empirical question that can only be assessed in future work, and our study aims primarily to improve empirical considerations of bundling.

## Conclusions

The development of ENDS policies in U.S. states has been temporally and geographically uneven. Yet, heading into the third decade of the 21^st^ century, states increasingly implemented a robust complement of policies designed to intervene on ENDS. Many states that had initially been slow to implement policies had caught up, and as a result, ENDS policies now increasingly establish the framework for preventing youth consumption while also permitting access for adult smokers who seek to reduce or cease combustible tobacco consumption. While the clustering of policies in bundles during implementation permits efficient enactment of public policy, it also has important methodological implications for analysis, which should be considered in ENDS policy evaluation.
